# Detection of tick-borne pathogens in blood-fed ticks from animals across nine Asian countries

**DOI:** 10.1128/spectrum.02449-24

**Published:** 2025-02-04

**Authors:** Hye-Ryung Byun, Mi-Sun Rieu, Seong-Ryeong Ji, Hyun-Young Nam, Seulgi Seo, Chang-Yong Choi, Bui Khanh Linh, Hien Le Thanh, Morakot Kaewthamasorn, Ana Sahara, Remil L. Galay, Shang-Lin Wang, Tuvshinjargal Erdenechimeg, Nyambayar Batbayar, Shin Matsui, Noritomo Kawaji, Muhammad Avais, Joon-Seok Chae

**Affiliations:** 1 Laboratory of Veterinary Internal Medicine, BK21 FOUR Future Veterinary Medicine Leading Education and Research Centre, Research Institute for Veterinary Science, College of Veterinary Medicine, Seoul National University, Seoul, South Korea; 2 School of Biological Sciences, College of Natural Sciences, Seoul National University, Seoul, South Korea; 3 Research Institute of Agriculture and Life Sciences, College of Agriculture and Life Sciences, Seoul National University, Seoul, South Korea; 4 Department of Agriculture, Forestry, and Bioresources, College of Agriculture and Life Sciences, Seoul National University, Seoul, South Korea; 5 Vietnam National University of Agriculture, Hanoi, Vietnam; 6 Department of Infectious Diseases and Veterinary Public Health, Faculty of Animal Science and Veterinary Medicine, Nong Lam University, Ho Chi Minh, Vietnam; 7 Veterinary Parasitology Research Unit, Faculty of Veterinary Science, Chulalongkorn University, Bangkok, Thailand; 8 Department of Parasitology, Faculty of Veterinary Medicine, Universitas Gadjah Mada, Yogyakarta, Indonesia; 9 Department of Veterinary Paraclinical Sciences, College of Veterinary Medicine, University of the Philippines Los Baños, Laguna, Philippines; 10 Institute of Veterinary Clinical Sciences, School of Veterinary Medicine, National Taiwan University, Taipei, Taiwan; 11 Wildlife Science and Conservation Center of Mongolia, Ulaanbaatar, Mongolia; 12 School of Biological Sciences, Tokai University, Sapporo, Hokkaido, Japan; 13 Hokkaido Research Center, Forestry and Forest Products Research Institute, Sapporo, Hokkaido, Japan; 14 Department of Veterinary Medicine, Faculty of Veterinary Science, University of Veterinary and Animal Sciences, Lahore, Pakistan; Central Texas Veterans Health Care System, Temple, Texas, USA

**Keywords:** ticks, tick-borne pathogens, NGS, Asia, environmental microbiology, animals

## Abstract

Climate change, land development, and increased outdoor human activity have increased the prevalence and distribution of tick-borne pathogens, causing public health issues. Asia is a pivotal region of emerging infectious diseases caused by zoonotic disease. Therefore, this study aimed to construct effective surveillance systems and establish preventive strategies against novel tick-borne diseases. Next-generation sequencing (NGS) was performed to detect tick-borne pathogens from animal blood-fed ticks. Ticks (*n* = 261) were collected from different animals across nine Asian countries between 2022 and 2023. Five genera of adults and nymphal ticks were used, namely, *Amblyomma*, *Haemaphysalis*, *Hyalomma*, *Rhipicephalus*, and *Ixodes*. The animals from which the ticks were collected were wild, domestic animals, birds, and reptiles, living in the natural environment. After NGS, clean reads of 18,382,262–30,460,619 DNA viruses, 22,744,384–32,400,471 RNA viruses, 134,506–286,282 16S rRNA of bacteria, and 147,154–352,826 18S rRNA of protozoa were obtained from nine Asian countries. After analysis of the species, 28 RNA, 13 DNA viruses, 16 bacteria, and 4 protozoa were detected. Several tick-borne pathogens, including those in families that cause zoonotic diseases, such as *Phenuiviridae*, *Nairoviridae*, *Rickettsiaceae*, and *Borreliaceae*, were identified. This study is distinctive in that it involved cooperative studies with nine Asian countries to prevent the spread of tick-borne diseases. The results suggest that pathogens, which were detected in animal blood-fed ticks, can circulate in animals and may be transmitted to humans. In addition, this study can provide a basis for effective surveillance systems to prevent novel pathogens.

**IMPORTANCE:**

Surveillance systems against novel tick-borne diseases are significant for global health. Climate and other environmental changes have contributed to an expanding range of ticks and tick-borne diseases. Areas in Asia constitute key areas of emerging infectious diseases. Through analysis of blood-fed ticks, collected from various animals living in the natural environment, we suggest that tick-borne pathogens may harbor animals and environment and have potential risk of transmission in humans. Understanding the distribution of tick-borne pathogens requires cooperative studying and, thus, can construct standardized surveillance systems.

## INTRODUCTION

Ticks are obligatory hematophagous ectoparasites and are considered a major worldwide health concern, the incidence of which is increasing owing to climate change and anthropogenically induced changes (1). Recent studies suggest that these changes may affect tick habitat suitability for the colonization of new areas and increase the spillover of emerging zoonotic pathogens, including tick-borne pathogens (2, 3). Ticks are divided into three families, *Ixodidae* (hard ticks), *Argasidae* (soft ticks), and *Nuttalliellidae*, with *Nuttalliella namaqua* being the only species in the *Nuttalliellidae* family (4). Several hard ticks have four different life stages: eggs, larvae, nymphs, and adults. Among the vertical transmission routes of tick-borne viruses, transstadial transmission is a sequential process that is vital for tick survival, whereas transovarial transmission occurs when viruses are transmitted from an infected tick to its offspring (5).

Likewise, with the expansion of tick populations and increasing incidence of tick-borne diseases, Asia is a region where various tick-borne pathogens have been identified and considered key areas for identifying infectious diseases caused by zoonotic pathogens (6, 7). Tick-borne pathogens can be transmitted to vertebrate hosts through various routes, especially birds, which may transport tick-borne viruses by amplifying the host through infection with tick-borne pathogens that can infect their parasitizing ticks, transmitting the pathogens to subsequent hosts (8). The tick infestation level can differ depending on the characteristics of the birds. Although resident birds may be more heavily infested with ticks, migratory birds play a more pivotal role in the long-distance dispersal of ticks (9). Ticks harbor various pathogens that may cause morbidity and mortality in humans and animals. Therefore, to identify potential candidates for explaining the unknown etiology of diseases, an established system for predicting outbreaks using advanced technologies is crucial (10).

Next-generation sequencing (NGS) offers rapid and high-throughput identification of both DNA and RNA libraries and is widely used in virome studies to identify novel or potential viral pathogens (11). This technology provides extensive preliminary data analysis for detecting pathogens in pooled tick samples and can subsequently be used for species confirmation and characterization via conventional methods paired with sequencing (12). Several metagenomic studies have revealed unknown pathogenic or novel viruses in various pooled ixodid ticks, and many tick-borne viruses have been identified in multiple studies (13–15). Although several metagenomic studies have demonstrated the presence of viruses in ticks, additional surveys of pathogens from other species by countries could reveal effective strategies against novel tick-borne pathogens and protect global public health.

This study aimed to analyze tick-borne pathogens, including viruses, bacteria, and protozoa, distributed in Asia from animal blood-fed ticks. Based on these results, we propose the possibility that tick-borne pathogens circulate in animals in each country and provide the basis for effective strategies for surveillance systems.

## RESULTS

### Ticks identified

In total, 261 ixodid ticks belonging to 13 species were used. In Indonesia, 34 ticks were used, and the most common tick species *Haemaphysalis bispinosa* (*n* = 17), *Amblyomma gervaisi* (*n* = 4), and *Amblyomma varanense* (*n* = 9) were collected only from Indonesia. In Japan, 36 ticks were used. *Ixodes persulcatus* (*n* = 29) was the most common genus collected, and it was also found in Mongolia (*n* = 26). In Pakistan, 22 ticks were used: *Hyalomma anatolicum* (*n* = 10), *Rhipicephalus microplus* (*n* = 10), *Rhipicephalus sanguineus* (*n* = 1), and *Hyalomma* spp. (*n* = 1).

In the Philippines, 20 ticks were used: *R. microplus* (*n* = 14) and *R. sanguineus* (*n* = 6). In the ROK, 45 ticks were used: *Haemaphysalis concinna* (*n* = 19) and *Haemaphysalis longicornis* (*n* = 19) were the most common. In Taiwan, 20 ticks were used: *Haemaphysalis hystricis* (*n* = 12) was the most common tick. In Thailand, 20 *R*. *microplus* ticks were used. In Vietnam, 38 ticks were used: *R. microplus* (*n* = 20), *R. sanguineus* (*n* = 7), and *H. bispinosa* (*n* = 11) (Table 1).

**TABLE 1:** Summary of ticks and host animals in each pooled sample across nine Asian countries

Classification	Countries	Pooling name	Tick species (no. of ticks)	Host animals	Developmental stages of ticks	No. of ticks
Mammals	Republic of Korea	ROK	*Haemaphysalis longicornis* (19)	Korean water deer, raccoon dog, Siberian roe deer	Adult, nymph	20
			*Haemaphysalis* spp. (1)	Korean water deer	Adult	
	Indonesia	IN	*Haemaphysalis bispinosa* (17)	Cattle, sheep	Adult, nymph	21
			*Rhipicephalus microplus* (3)	Cattle	Adult	
			*Rhipicephalus sanguineus* (1)	Dog	Adult	
	Pakistan	PA	*Rhipicephalus microplus* (10)	Cattle, buffalo	Adult	22
			*Hyalomma anatolicum* (10)	Cattle, buffalo, goat	Adult	
			*Rhipicephalus sanguineus* (1)	Dog	Adult	
			*Hyalomma* spp. (1)	Rabbit	Nymph	
	Philippines	PH	*Rhipicephalus microplus* (14)	Cattle	Adult	20
			*Rhipicephalus sanguineus* (6)	Dog	Adult	
	Taiwan	TA	*Haemaphysalis hystricis* (12)	Dog	Adult	20
			*Rhipicephalus sanguineus* (8)	Dog	Adult	
	Thailand	TH	*Rhipicephalus microplus* (20)	Cattle, buffalo	Adult	20
	Vietnam	VI	*Rhipicephalus microplus* (20)	Cattle, goat	Adult, nymph	38
			*Rhipicephalus sanguineus* (7)	Dog	Adult	
			*Haemaphysalis bispinosa* (11)	Goat	Adult, nymph	
Birds	Republic of Korea	ROK	*Haemaphysalis concinna* (19)	Little bunting, black-faced bunting, olive-backed pipit, Pallas’s reed bunting	Nymph	25
			*Haemaphysalis flava* (3)	Black-faced bunting, yellow-browed bunting	Nymph	
			*Ixodes nipponenesis* (2)	Black-faced bunting, yellow-browed bunting	Nymph	
			*Haemaphysalis longicornis* (1)	Olive-backed pipit	Nymph	
	Japan	JA	*Ixodes persulcatus* (29)	Gray bunting, Japanese thrush, great spotted woodpecker, red-flanked bluetail, Japanese tit, masked bunting, Siberian blue robin	Nymph	36
			*Ixodes nipponensis* (4)	Gray bunting, black-faced bunting	Nymph	
			*Haemaphysalis flava* (2)	Gray bunting, black-faced bunting	Nymph	
			*Ixodes turdus* (1)	Eurasian wren	Nymph	
	Mongolia	MO	*Ixodes persulcatus* (26)	Thick-billed warbler, little bunting, chestnut bunting, eastern yellow wagtail, Pallas’s grasshopper warbler, black-faced bunting, yellow-browed warbler, Chinese bush warbler, dusky warbler, red-breasted flycatcher, Pallas’s leaf warbler	Nymph	26
Reptiles	Indonesia	IN	*Amblyomma varanense* (9)	Asian water monitor, snakes	Adult	13
			*Amblyomma gervaisi* (4)	Asian water monitor, snakes	Adult	
Total	Nine countries	Nine pools	13 tick species	33 animal species	162 adult ticks, 99 nymph ticks	261

### Ticks collected from animal species

The animals from which the ticks were collected included mammals, birds, and reptiles.

By country, the animals included were Indonesia, cattle, sheep, dogs, and Asian water monitors (*Varanus salvator*) and snakes; Japan, gray bunting (*Emberiza variabilis*), Japanese thrush (*Turdus cardis*), great spotted woodpecker (*Dendrocopos major*), red-flanked bluetail (*Tarsiger cyanurus*), Japanese tit (*Parus minor*), masked bunting (*Emberiza spodocephala*), Siberian blue robin (*Luscinia cyane*), black-faced bunting (*Emberiza personata*), and Eurasian wren (*Troglodytes troglodytes*); Mongolia, Thick-billed warbler (*Acrocephalus aedon*), little bunting (*Emberiza pusilla*), chestnut bunting (*Emberiza rutile*), Eastern yellow wagtail (*Motacilla tschutschensis*), Pallas’s grasshopper warbler (*Locustella certhiola*), black-faced bunting, yellow-browed warbler (*Phylloscopus inornatus*), and Chinese bush warbler (*Locustella tacsanowskia*). Wild animals were collected only from the ROK, birds were collected in Japan, Mongolia, and the ROK, and only reptiles were collected in Indonesia (Table 1).

### Overall results

**Fig 1:**
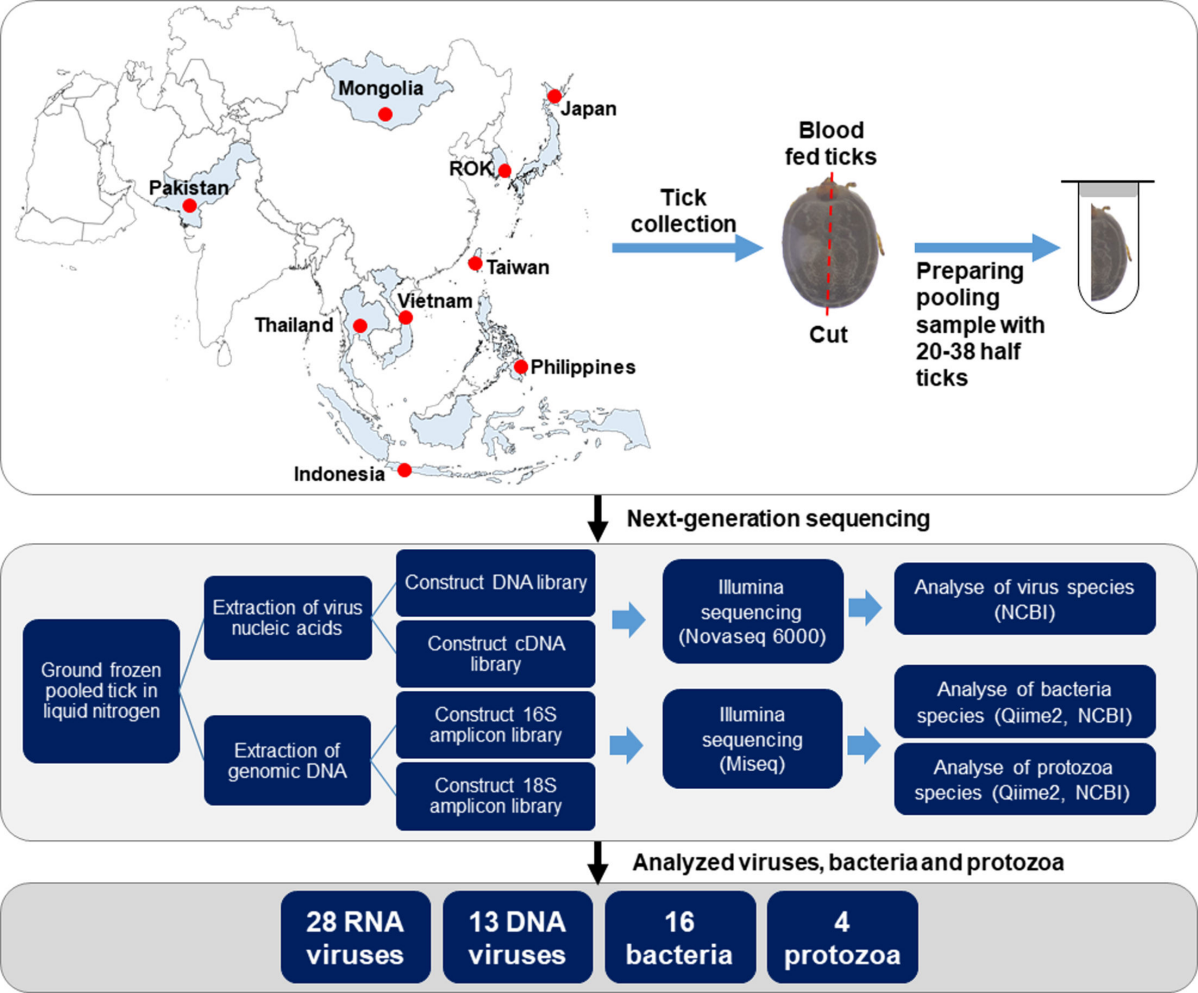
Overview of the analysis of pathogens from blood-fed ticks across nine Asian countries using *de novo* assembly via next-generation sequencing. In each pooled sample, 20–38 half of the blood-fed ticks were used. Red dots indicate most tick collection sites. The map was sourced from the data center of the National Geographic Information Institute in the Republic of Korea (https://www.ngii.go.kr/eng/content.do?sq=303).

**Fig 2:**
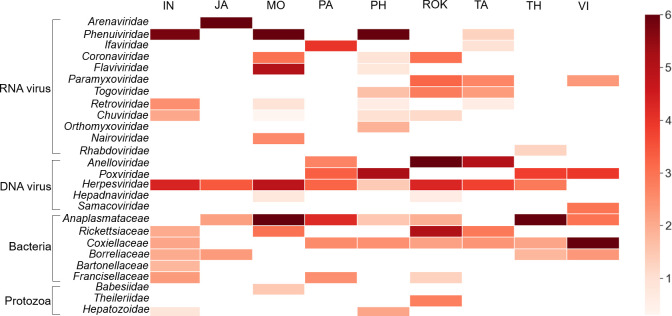
The distribution of 26 viruses, bacteria, and protozoan families from nine pooled tick samples obtained from nine Asian countries based on annotated reads. IN, Indonesia; JA, Japan; MO, Mongolia; PA, Pakistan; PH, the Philippines; ROK, the Republic of Korea; TA, Taiwan; TH, Thailand; VI, Vietnam.

After *de novo* assembly sequencing, a total of 20 RNA viruses, 5 DNA viruses, 6 bacteria, and 3 protozoa were identified from blood-fed ticks from nine Asian countries (Fig. 1). At the family level, 26 families and species level, 28 RNA viruses, 13 DNA viruses according to Illumina NovaSeq, 16 bacteria and 4 protozoa according to Illumina MiSeq were identified from animal blood-fed ticks (Tables 2 and 3; Fig. 2). Viral data were analyzed via NCBI BLASTN, and bacterial and protozoan data were analyzed via Qiime2 and NCBI BLASTN data. Plant viruses and phages were excluded from this study.

**TABLE 2:** Characterization of viruses identified from blood-fed ticks by next-generation sequencing

Type	Detected countries	Family	Species	No. of reads	Identity (%, BLASTN)	GenBank accession number
RNA virus	Japan	*Arenaviridae*	Guanarito mammarenavirus	1,284,909	97.6	KU746283
	Philippines	*Phenuiviridae*	Uukuvirus lihanense	452,279	96.7	MW721920
	Indonesia		Okutama tick virus	106,191	91.2	OQ513686
	Taiwan		Brown dog tick phlebovirus 2	10	96	OM326753
	Indonesia			6	89.2	OM326753
	Mongolia		Mukawa phlebovirus	88	87.9	ON408118
	Mongolia		Sara tick phlebovirus	9	99.4	ON408148
	Taiwan		Bangxi Phenu tick virus 1	2	85.8	ON746499
	Pakistan	*Iflaviridae*	Iflavirus HdromIV	9,757	88.9, 91.4	BK012003, MZ567077
	Taiwan		Sacbrood virus	9	100	MN528599
	ROK	*Coronaviridae*	SARS-related coronavirus	1,077	93.5, 100	OQ297705, MT671829
	Mongolia			1,035	93.6	OQ297705
	Philippines			8	100	OV178357
	Mongolia	*Flaviviridae*	Pestivirus B	501	88.6, 89.7	AF268174, AF104025
	Mongolia		Pestivirus A	200	85.8	LT907991
	Philippines		Trinbago virus	6	97.9	MN025505
	ROK	*Paramyxoviridae*	Human respirovirus 3	1,604	100	OP821798
	Taiwan			447	100	OP821798
	Vietnam			209	100	OP821798
	Philippines	*Togaviridae*	Chikungunya virus	44	89.3	OL343610
	ROK		Semliki forest virus	661	100	Z48163
	Taiwan			182	99.5	Z48163
	Indonesia	*Retroviridae*	Human endogenous retrovirus H	168	96.5	AJ289710
	Mongolia		Human immunodeficiency virus 1	8	85	MZ766785
	Philippines		Jaagsiekte sheep retrovirus	4	95.6	AF357971
	Taiwan		Equine infectious anemia virus	4	100	M16575
	Indonesia		Human endogenous retrovirus K	2	95.3	Y18890
	Indonesia	*Chuviridae*	Changping mivirus	118	99.2	MN095545
	ROK			14	99.3	MN095545
	Philippines			10	100	MN095545
	Mongolia		Lesnoe mivirus	2	100	OP628502
	Philippines	*Orthomyxoviridae*	Xiangxi ortho tick virus 1	78	86.8	ON746462
	Mongolia	*Nairoviridae*	Beiji nairovirus	68	99.1, 98.7, 98.6	ON408097, ON408104, MG880120
	Mongolia		Gakugsa tick virus	6	98.7	ON811927
	Thailand	*Rhabdoviridae*	Wuhan tick virus 1	19	99.3	MN095546
	Pakistan	Unclassified	Hubei tick virus 1	1,011	72.3	OQ513642
DNA virus	ROK	*Anelloviridae*	Paguma larvata torque teno virus	2,324,503	77.5	NC_076176
	Taiwan		Torque teno Tadarida brasiliensis virus 2	62,330	90.8	OL704857
	Pakistan			488	89.2	OL704857
	Philippines	*Poxviridae*	ORF virus	144,903	91.1	HM133903
	Vietnam			8,838	82.8	HM133903
	Thailand			6,783	83.3	HM133903
	Pakistan			2,056	84.3	HM133903
	Mongolia	*Herpesviridae*	Cercopithecine betaherpesvirus 5	74,397	86.4, 87.1	AF191073, AF065755
	Indonesia			21,761	91.1	AF191073
	ROK			19,369	88.4, 88.8, 89.1	AF065756, AF065755, AF191073
	Taiwan			5,970	89, 90.9	AF065755, AF065756
	Japan			2,530	91.0	AF191073
	Pakistan			1,701	87.6, 97.4	AF065755, AF191073
	Thailand			733	85.3	AF191073
	Philippines			28	80.2	AF065755
	Japan		Human betaherpesvirus 5	9	100	MN920393
	Mongolia		Human alphaherpesvirus 1	14	99.3	AB618031
	Taiwan			2	99.6	FJ593289
	Mongolia		Human gammaherpesvirus 4	8	88.4, 95.6	MH590507, MH590409
	ROK			6	96.1	MH590571
	Mongolia		Human gammaherpesvirus 8	8	100	MK733609
	Pakistan		Equid alphaherpesvirus 1	10	100	LC193725
	Indonesia		Gallid alphaherpesvirus 1	30	100	KX165321, KY423284
	ROK		Macacine betaherpesvirus 3	2	100	MN437483
	Mongolia	*Hepadnaviridae*	Woodchuck hepatitis virus	6	99.5	J04514
	ROK			4	100	J04514
	Vietnam	*Samacoviridae*	Porcine-associated porprismacovirus	905	88.3	MH500309

**TABLE 3:** Characterization of the identified 16S rDNA gene of bacteria and 18S rDNA of protozoa from blood-fed ticks by next-generation sequencing

Type	Detected countries	Family	Species	No. of reads	Identity (%, Qiime2, BLASTN)	GenBank accession number
Bacteria (16S)	Vietnam	*Anaplasmataceae*	*Anaplasma phagocytophilum*	952	98.2	NR_044762
	Mongolia			852	99.8	NR_044762
	Thailand			255	98.9	NR_044762
	Pakistan			87	98.6	NR_044762
	Thailand		*Anaplasma odocoilei*	150	99.3	NR_118489
	ROK			88	98	NR_118489
	Thailand		*Ehrlichia ewingii*	237	99	NR_044747
	Pakistan			210	98.9	NR_044747
	Mongolia			83	90.7	NR_044747
	Philippines			33	99.1	NR_044747
	Mongolia		*Ehrlichia muris*	955	99.7	NR_121714
	Japan			156	99.3	MN658723
	Mongolia	*Rickettsiaceae*	*Rickettsia slovaca*	1,698	99.8	NR_179179
	Taiwan			785	99.8	NR_179179
	Indonesia			2	99.4	NR_179179
	Indonesia		*Rickettsia bellii*	107	99.8	NR_074484
	ROK		*Rickettsia tamurae*	552	99.3	NR_134842
	ROK		*Rickettsia japonica*	241	99.8	NR_074459
	Vietnam	*Coxiellaceae*	*Coxiella burnetii*	462	96.4	NR_104916
	Philippines			295	96.1	NR_104916
	Taiwan			247	97.2	NR_104916
	ROK			149	94.8	NR_104916
	Vietnam		*Coxiella cheraxi*	8,510	94.2	NR_116014
	Pakistan			355	94.8	NR_116014
	Thailand			131	94	NR_116014
	Indonesia			130	94	NR_116014
	Japan	*Borreliaceae*	*Borrelia puertoricensis*	305	98.1	NR_181316
	Vietnam		*Borrelia hermsii*	222	98.3	NR_102957
	Indonesia		*Borrelia garinii*	101	99.8	KY312013
	Thailand		*Borrelia miyamotoi*	63	98.2	NR_025861
	Indonesia	*Bartonellaceae*	*Bartonella schoenbuchensis*	67	99.5	NR_025410
	Pakistan	*Francisellaceae*	*Francisella hispaniensis*	309	98.7	NR_116944
	Indonesia			209	98.7	NR_116944
	ROK			21	98.9	NR_116944
Protozoa (18S)	Mongolia	*Babesiidae*	*Babesia microti*	29	99.7	MK609547
	ROK	*Theileriidae*	*Theileria luwenshuni*	618	100	MN626389
	Indonesia	*Hepatozoidae*	*Hepatozoon ophisauri*	7	100	MN723845
	Philippines		*Hepatozoon canis*	130	99.8	MK830996

### Viral results

After Illumina NovaSeq 6000 sequencing, 18,382,262–30,460,619 DNA and 22,744,384–32,400,471 RNA clean viral reads were obtained from each country. By country, in Indonesia, there were 21,284,226 DNA and 29,164,147 RNA viral reads; in Japan, 21,897,783 DNA and 22,744,384 RNA reads; in Mongolia, 27,881,708 DNA and 32,299,656 RNA; in Pakistan, 19,454,884 DNA and 31,520,672 RNA reads; in the Philippines, 26,095,061 DNA and 32,400,471 RNA reads; in the ROK, 30,460,619 DNA and 32,001,059 RNA; in Taiwan, 19,988,226 DNA and 25,364,519 RNA reads; in Thailand, 18,382,262 DNA and 29,421,110 RNA reads; and in Vietnam, 27,206,663 DNA and 32,347,593 RNA reads were obtained.

These viral reads were annotated to 17 families, with one unclassified family, and 28 RNA and 13 DNA virus species were identified. Viruses belonging to the families *Arenaviridae*, *Chuviridae*, *Coronaviridae*, *Flaviviridae*, *Iflaviridae*, *Nairoviridae*, *Orthomyxoviridae*, *Paramyxoviridae*, *Phenuiviridae*, *Retroviridae*, *Rhabdoviridae*, and *Togaviridae* possessed RNA viral genomes, whereas those belonging to the families *Anelloviridae*, *Hepadnaviridae*, *Herpesviridae*, *Poxviridae*, and *Smacoviridae* possessed DNA viral genomes.

Among the viruses identified via BLASTN, the most abundant read was from the Guanarito mammarenavirus (GTOV), which had 1,284,909 viral reads and was detected in ticks from Japan. After analysis of the virus via BLASTN, 97.6% identity was obtained with the reference GenBank ID (KU746283) (Table 2). By country, Mongolia had more viruses detected than other regions. In the pooled Mongolian tick sample, all ticks were collected from the birds.

### Bacterial results

After Illumina MiSeq sequencing, 158,600–286,282 of the 16S rRNA bacteria were obtained from each country. By country, in Indonesia, there were 204,402 reads; in Japan, there were 187,606 reads; in Mongolia, there were 266,586 reads; in Pakistan, there were 216,846 reads; in the Philippines, there were 235,516 reads; in the ROK, there were 286,282 reads; in Taiwan, there were 224,848 reads; in Thailand, there were 158,600 reads; and in Vietnam, there were 256,068 reads.

A total of six families and 16 species of 16S bacteria were identified via Qiime2 and BLASTN. The identified 16S bacteria belonged to the *Anaplasmataceae*, *Bartonellaceae*, *Borreliaceae*, *Coxiellaceae*, *Francisellaceae*, and *Rickettsiaceae* families. Among the 16 identified bacteria, the tick-borne bacteria were *Anaplasma phagocytophilum*, *Ehrlichia ewingii*, *Ehrlichia muris*, *Rickettsia slovaca*, *Rickettsia bellii*, *Rickettsia tamurae*, *Rickettsia japonica*, and *Coxiella burnetii*.

In this study, *Coxiella cheraxi* was the longest read, with 8,510 reads detected from ticks in Vietnam and 94.2% identity to the reference GenBank accession number (NR_116014) (Table 3).

### Protozoa results

After Illumina MiSeq sequencing, 197,840–352,826 18S rRNA protozoal reads were obtained from each country. By country, in Indonesia, 253,784 reads; in Japan, 174,786 reads; in Mongolia, 295,884 reads; in Pakistan, 197,840 reads; in the Philippines, 275,434 reads; in the ROK, 352,826 reads; in Taiwan, 225,560 reads; in Thailand, 196,498 reads; and in Vietnam, 230,306 reads were obtained.

Analysis of the Qiime2 and BLASTN data revealed 18S protozoa belonging to the *Babesiidae*, *Theileriidae*, and *Hepatozoidae* families.

18S protozoan species were identified from only four countries, namely, Indonesia, Mongolia, the Philippines, and the ROK. Four species, *Babesia microti*, *Theileria luwenshuni*, *Hepatozoon ophisauri*, and *Hepatozoon canis*, were identified; *Theileria luwenshuni* in the ROK, *Hepatozoon canis* in the Philippines, *Babesia microti* in Mongolia, and *Hepatozoon ophisauri* in Indonesia (Table 3).

## DISCUSSION

This study performed a metagenomic analysis of pooled ticks using *de novo* assembly NGS technologies and revealed diverse pathogens, including viruses, bacteria and protozoa, from animal blood-fed ticks.

Five zoonotic viruses, namely, GTOV, beiji nairovirus (BJNV), chikungunya virus (CHIKV), ORF virus (ORFV), and semliki forest virus (SFV), were identified in this study. Among them, BJNV, which clusters into *Nairoviridae*, has not been well studied or reported to be pathogenic; however, recent studies have examined tick-borne febrile illness in humans (16). In *Arenaviridae*, GTOV, commonly known as a rodent-borne virus, was found in ticks from birds in Japan as the etiological agent of Venezuelan hemorrhagic fever, for which the overall incidence was ≥33.3% (17). The reservoir host of GTOV, known as *Zygodontomys brevicauda*, causes no lethal infection with GTOV; however, it causes chronic viremia with persistent and long-term shedding of viral particles in oropharyngeal secretions and urine (17). Illumina NovaSeq generated 1,284,909 GTOV reads from pooled Mongolian samples. Pooled Mongolian samples, including blood-fed ticks, were collected from birds; thus, this virus may be circulating in birds and be detected in blood-fed ticks. In the *Poxviridae* family, ORFV strains in Taiwan, Thailand, Pakistan, and the Philippines are highly contagious infections in sheep and goats (18). The transmission routes of these viruses to humans usually occur through direct or indirect contact with infected sheep and goats, but transmission via ticks has not been determined. Although CHIKV and SFV constitute a group of mosquito-borne viruses, they were identified in blood-fed ticks in this study. CHIKV was found in cattle blood-fed ticks in the Philippines, and SFV was found in wild animals and dog blood-fed ticks in the ROK and Taiwan. Given that the subgenus *Aedes*, which prefers to feed on cattle, can facilitate the circulation of CHIKV and wild animals, which are the natural hosts of SFV, this study may have confirmed that the viruses circulated in animals via tick analysis (19, 20)

Ticks contain abundant commensal and mutualistic microbes that directly release molecules that inhibit the growth of a tick-borne pathogen competitor or promote tick-borne pathogen development during immunosuppression. Therefore, based on the features of tick-microbial interactions, nonpathogenic microorganisms may facilitate, inhibit, or limit tick-borne pathogen transmission (21). Well-known examples of nonpathogenic microorganisms are the *Rickettsia*, *Francisella*, and *Coxiella* genera, which are traditionally known as highly virulent vertebrate pathogens (21).

This study identified several species of these bacteria, such as *Rickettsia slovaca*, *Francisella hispaniensis*, and *Coxiella cheraxi*, in blood-fed ticks, which provides insight into the presence of phenotypic diversity and requires additional research on symbiotic forms adapted to hosts and ticks.

Zoonotic tick-borne bacteria, such as *Anaplasma phagocytophilum*, *Coxiella burnetii*, and *Ehrlichia ewingii*, cause anaplerosis, Q fever, and ehrlichiosis, respectively; these bacteria are widely distributed worldwide and cause various clinical manifestations in humans (22). In protozoa, *Babesia microti*, which is the main etiologic agent of human babesiosis and is maintained in nature through an enzootic cycle between ticks and small mammals (23) was detected in birds from Mongolia. *Babesia microti* was discovered in blood-fed larval ticks on several species of birds in Europe, and white-footed mice (*Peromyscus leucopus*) are a predominant reservoir host for *Babesia microti* (24). The results of the present study suggest that tick-borne pathogens may circulate in animals such as mammals, birds, and reptiles and that these animals are at risk of transmitting disease to humans. For instance, several viruses were detected in *Haemaphysalis* and *Ixodes* ticks collected from migratory and resident birds. In a recent study, severe fever with thrombocytopenia syndrome virus (*Bandavirus dabieense*) was newly detected in the nymph *Haemaphysalis concinna* and *Ixodes turdus* collected from two migratory birds, black-faced bunting (*Emberiza spodocephala*) and olive-backed pipit (*Anthus hodgsoni*), respectively, in the ROK (25). These birds were captured on western islands in the spring migratory season, which strongly suggests that ticks migrated northward into the ROK after sea-crossing flights. Moreover, songbirds are carriers of ticks and tick-borne pathogens, which can lead to long distances of bird migration (26). Most migratory songbirds live on East Asian flyways; in particular, East Asia habitats provide connecting regions with stopover sites for migrating songbirds to move between temperate breeding and tropical wintering for survival (27). Therefore, such zoonotic viruses can be transmitted across the board via migratory birds. The current study has several limitations. First, ticks were pooled from only nine Asian countries, which does not reflect tick diversity or the abundance of pathogens in the entire Asian region. Second, this study focused on identifying novel tick-borne pathogens, and data on their genetic characteristics are lacking. Third, ticks were collected from birds in only the ROK, Japan, and Mongolia, limiting the ability to reveal the relationship between migratory birds and tick-borne pathogens. Fourth, the presence of viral DNA or RNA in a tick does not directly confirm that the tick is a biological vector for those viruses; rather, it may simply indicate that the tick is a mechanical vector. Finally, the pathogenicity of several identified pathogens in humans and animals has yet to be elucidated; thus, further investigations need to confirm the causal ability of these pathogens.

In summary, relevant and abundant pathogens may circulate worldwide using ticks as vectors. Future studies should focus on identifying pathogens that are possibly pathogenic to animals and humans. Moreover, preventive measures should be developed through collaborative research based on the genetic relationships of each virus and the One Health concept.

## MATERIALS AND METHODS

### Collection of ticks

Between April 2022 and August 2023, ticks were collected from mammals, birds, and reptiles in nine countries: Indonesia, Japan, Mongolia, Pakistan, the Philippines, the ROK, Taiwan, Thailand, and Vietnam. The primary collection sites in each country were accessible sites where ticks were abundantly distributed. Ticks were manually removed from the mammals on the farms and from pastures, from wild animals in the wildlife rescue centers and from reptiles in a snake slaughterhouse and zoos. Blood-fed ticks were collected from birds captured using mist nets between August 2022 and May 2023. The mist nets were opened from sunrise to sunset or from sunrise to early morning and checked every 30 min or 1 h, respectively, according to the bird-banding protocols of the stations. After a routine bird-banding procedure, which included species, age, and sex identification, attachment of a metal band for individual identification, and collection of morphological data, the entire head of each bird was searched for ticks.

### Tick identification

After collection, the ticks were identified and assessed based on their morphological features. Tick species were identified using Petri dishes and forceps with two stereomicroscopes (Olympus SZ microscope with WF20X/12 mm eyepieces, Japan; Olympus SZH10 research stereomicroscope with GWH10X-CD eyepieces, Japan). All ticks were identified using ice packs to minimize RNA degradation. Due to damage to their morphological structures when removed from the animals, unrecognized species were marked as “spp.”

### Tick pooling

Nine pooled halves of the ticks were prepared for NGS. The pooled samples were named with a letter indicating their country of origin: IN (Indonesia), JA (Japan), MO (Mongolia), PA (Pakistan), PH (Philippines), ROK (Republic of Korea), TA (Taiwan), TH (Thailand), or VI (Vietnam). For organized tick pooling, first, ticks were initially selected from each country to assess the differences in pathogens among ticks obtained from different countries. Second, for the specific analysis of tick-borne viromes, ticks were selected from host animals (mammals, birds, and reptiles). Third, ticks (*Amblyomma*, *Haemaphysalis*, *Hyalomma*, *Rhipicephalus*, and *Ixodes*) at different life stages (adults and nymphs) were selected. All the selected ticks were blood-fed ticks.

### Library construction

Viral nucleic acids were extracted from each ground tick sample using a QIAamp MinElute Virus Spin Kit (Qiagen, Hilden, Germany) according to the manufacturer’s instructions. Each pooled sample was prepared from the viral DNA libraries for Illumina sequencing using a QIAseq FX Single Cell DNA Library Kit (Qiagen) with viral DNA. Ribosomal RNA was removed from the viral RNA using the QIAseq FastSelect-RNA HMR Kit (Qiagen), followed by the preparation of complementary DNA libraries for Illumina sequencing with the QIAseq FX Single-cell RNA Library Kit (Qiagen).

The quality and concentration of the library were confirmed using the TapeStation HS D5000 ScreenTape system (Agilent Technologies, Santa Clara, CA, USA) and LightCycler 480 instrument (Roche, Basel, Switzerland), with library fragments exceeding 600 bp. The validated libraries were subsequently sequenced on a NovaSeq 6000 system (Illumina, San Diego, CA, USA) to generate 150 bp paired-end reads.

Following the manufacturer’s instructions, genomic DNA was extracted using a DNeasy Blood & Tissue Kit (Qiagen). To construct the 16S rRNA V3-V4 region of bacteria and the 18S rRNA region of the protozoan amplicon library, we used overhang PCR with KAPA HiFi Hotstart ready mix (Roche) and the Nextera XT DNA Sample Preparation Kit (Illumina). The sequences of primers used were as follows: 16S rRNA V3-V4 region forward 5′-CCTACGGGNGGCWGCAG-3′ and reverse 5′-GACTACHVGGGTATCTAATCC-3′ (28) and 18S rRNA forward 5′-GCCGCGGTAATTCCAGCTC-3′ and reverse 5′-CYTTCGYYCTTGATTRA-3′ (29). The quality and concentration of the library were verified using the TapeStation HS D5000 ScreenTape system (Agilent Technologies) and PicoGreen, with library fragments exceeding 550 bp. Sequencing was performed on the Illumina MiSeq platform to generate 300 bp paired-end reads.

### Data filtering

Adapter sequences were eliminated, and low-quality sequences (<Q30) were filtered out using Trim Galore (v.0.6.10) (https://github.com/FelixKrueger/TrimGalore). The *de novo* assembly SPAdes genome assembler (v.3.15.5) was used to trim reads assembled into a contig via the “--metaviral” pipeline for DNA and “--rnaviral” pipeline for RNA (30). The obtained contig (exceeding 100 bp) was annotated against the National Center for Biotechnology Information (NCBI) virus nucleotide database (https://ftp.ncbi.nlm.nih.gov/blast/db/) hits using BLASTN (v.2.13.0+), with rank = 1 and e-value = 1 × 10^−10^. BLASTX (v.2.13.0+) was used against the standard NCBI nonredundant protein sequence database with rank = 1 and e-value = 1 × 10^−5^ to identify duplicated data. The priority data selected from the viral contigs were >200 bp in length, with >90% coverage compared with that of reference sequences, and excluded phage and plant viruses. The adapter and low-quality sequences were eliminated from the 16S bacteria/18S protozoa (<Q30) using TrimGalore (v.0.6.10) (https://github.com/FelixKrueger/TrimGalore). Species were analyzed using the Qiime2 Sliva (https://www.arb-silva.de/), NCBI nucleotide (rank = 1, e-value ≤1e−10), and NCBI 16S databases.
